# Supplementary material for: The COVID-19 Vaccination Strategy in Brazil—A Case Study
Source: Epidemiologia (Basel). 2021 Aug 12;2(3):338–59. doi: 10.3390/epidemiologia2030026 (PMC9620893; doi:10.3390/epidemiologia2030026)
Supplement: Supplementary file 1 [file epidemiologia-02-00026-s001.zip › epidemiologia-1286880-supplementary.pdf]

epidemiologia

MDPI

Article

The COVID-19 Vaccination Strategy in Brazil — A Case Study

Llanos Bernardeau-Serra, Agathe Nguyen-Huynh, Lara Sponagel, Nathalia Sernizon Guimarães, Raphael Augusto Teixeira de Aguiar and Milena Soriano Marcolino

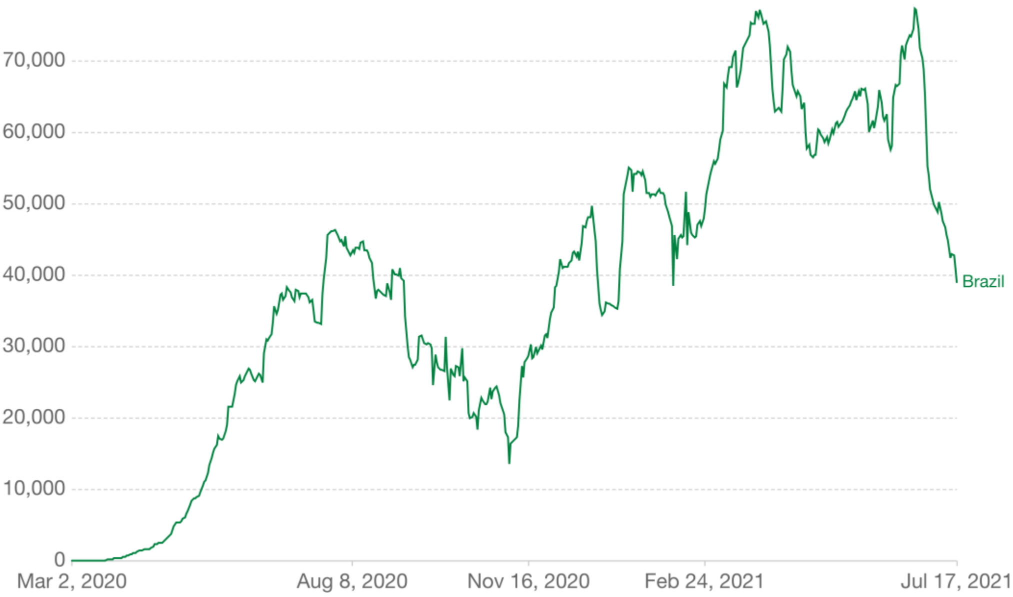

Figure S1. Seven-day average of daily new confirmed COVID-19 cases. Source: Ritchie H et al. "Coronavirus Pandemic (COVID-19)", 2020. *Published online at OurWorldInData.org.* Retrieved from: https://ourworldindata.org/coronavirus, accessed on 18 July 2021.

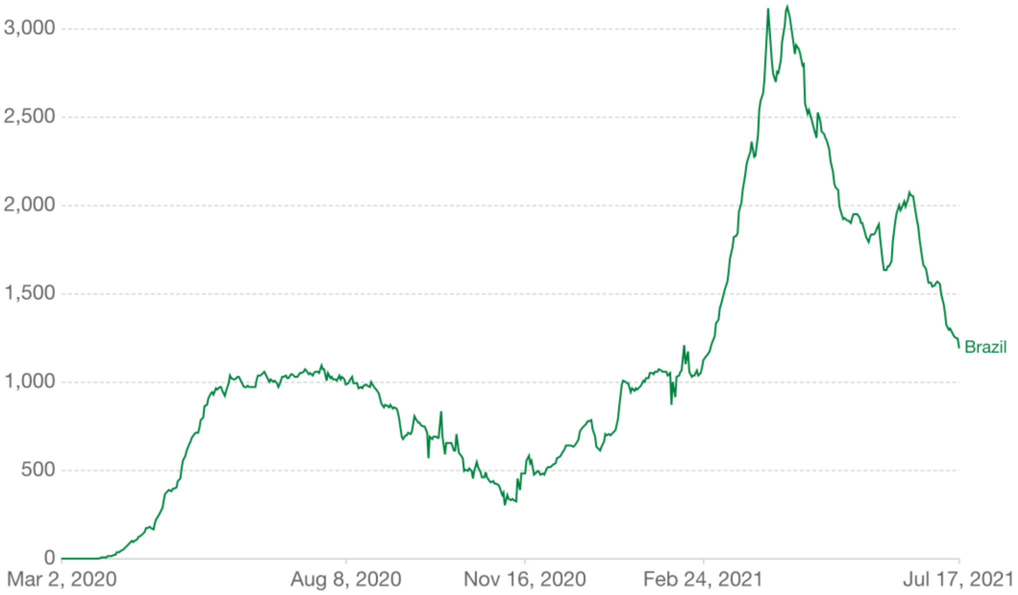

Figure S2. Seven-day average of daily new confirmed COVID-19 cases. "Coronavirus Pandemic (COVID-19)", 2020. *Published online at OurWorldInData.org*. Retrieved from: https://ourworldindata.org/coronavirus, accessed on 18 July 2021.

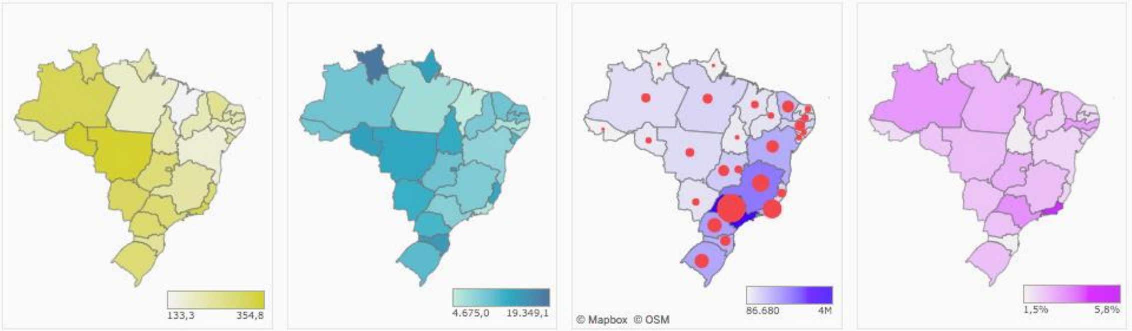

Figure S3. COVID-19 mortality rate per 100,000 inhabitants (**A**), incidence rate per 100,000 inhabitants (**B**), lethality rate per 100,000 inhabitants (C) and overall lethality (**D**) in each Region of Brazil as of 18 July 2021 Source: Conselho Nacional de Secretários de Saúde – CONASS. Painel CONASS COVID-19. Retrieved from: https://www.conass.org.br/painel-conasscovid19/, accessed on 18 July 2021.

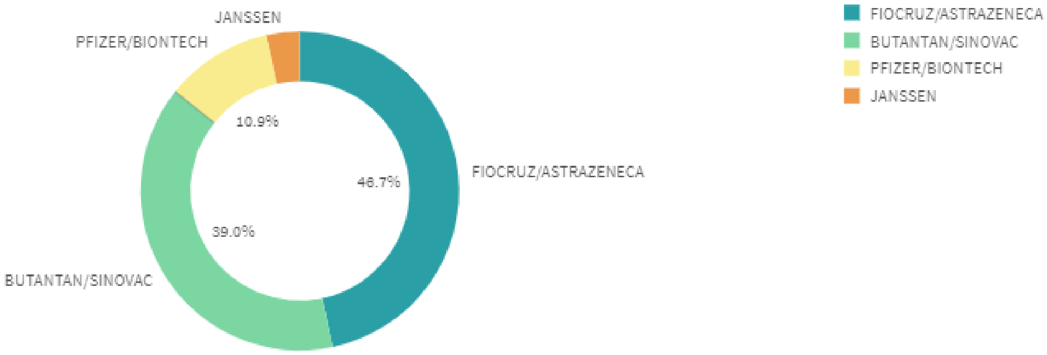

Figure S4. Applied doses according to producer as of 19 July 2021. Source: https://qsprod.saude.gov.br/extensions/DE-MAS_C19Vacina/DEMAS_C19Vacina.html, accessed on 18 July 2021.

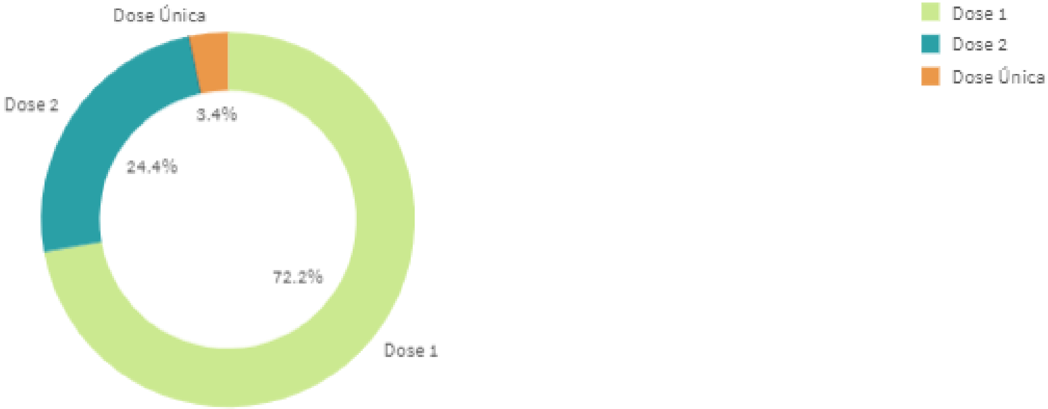

Figure S5. Rate of population who has received one dose (green, partially immunized), two doses (blue, immunized) or one dose of Janssen (orange, immunized) as of 19 of July 2021. Source: https://qsprod.saude.gov.br/extensions/DE-MAS C19Vacina/DEMAS C19Vacina.html, accessed on 18 July 2021.

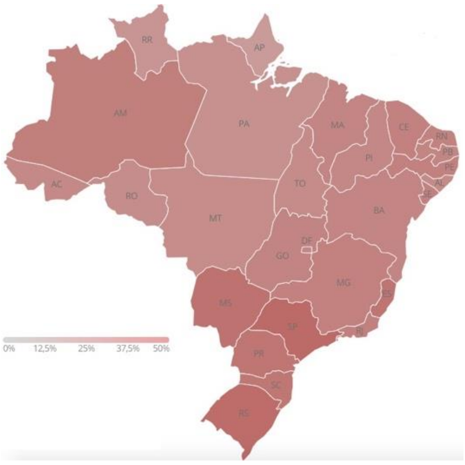

Figure S6. Percentage of the population who received the first Covid-19 vaccine shot as of 18 July 2021. Source: G1. Mapa da vacinação contra Covid-19 no Brasil. Retrieved from: https://especiais.g1.globo.com/bemestar/vacina/2021/mapa-brasil-vacina-covid, accessed on 18 July 2021.
